# Catheter ablation of uncommon epicardial accessory pathway sites: A single‐center case series

**DOI:** 10.1002/joa3.70102

**Published:** 2025-06-04

**Authors:** Haikal Balweel, Rifqi Rizkani Eri, Novaro Adeneur Tafriend, Sania Zahrani, Agus Harsoyo

**Affiliations:** ^1^ Gatot Soebroto Army Central Hospital Jakarta Indonesia; ^2^ Faculty of Medicine Universitas Indonesia Jakarta Indonesia

**Keywords:** catheter ablation, epicardial accessory pathway, SVT, Wolff‐Parkinson‐White syndrome

## Abstract

Uncommon sites of epicardial accessory pathways: coronary sinus, atrial appendage, aortic cusps.

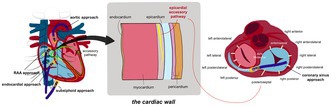

## 1 BACKGROUND

Accessory pathways (AP) are anomalous cardiac electrical connections that bypass the atrioventricular node, directly linking the atria and ventricles, skipping the decremental function of the AV node, which normally provides a necessary delay between atrial and ventricular depolarization.^1^ Most APs can be ablated from the endocardium. However, some may have epicardial components or be entirely epicardial, making them challenging to ablate. Certain uncommon epicardial AP sites can still be ablated from the endocardium or surrounding structures such as the vascular system, including the atrial appendage, coronary sinus, and aortic cusps. Here, we present three cases of uncommon epicardial AP sites ablated from the right atrial appendage, distal coronary sinus, and noncoronary cusp.

## 2 FIRST CASE: CORONARY SINUS APPROACH

**FIGURE 1:**
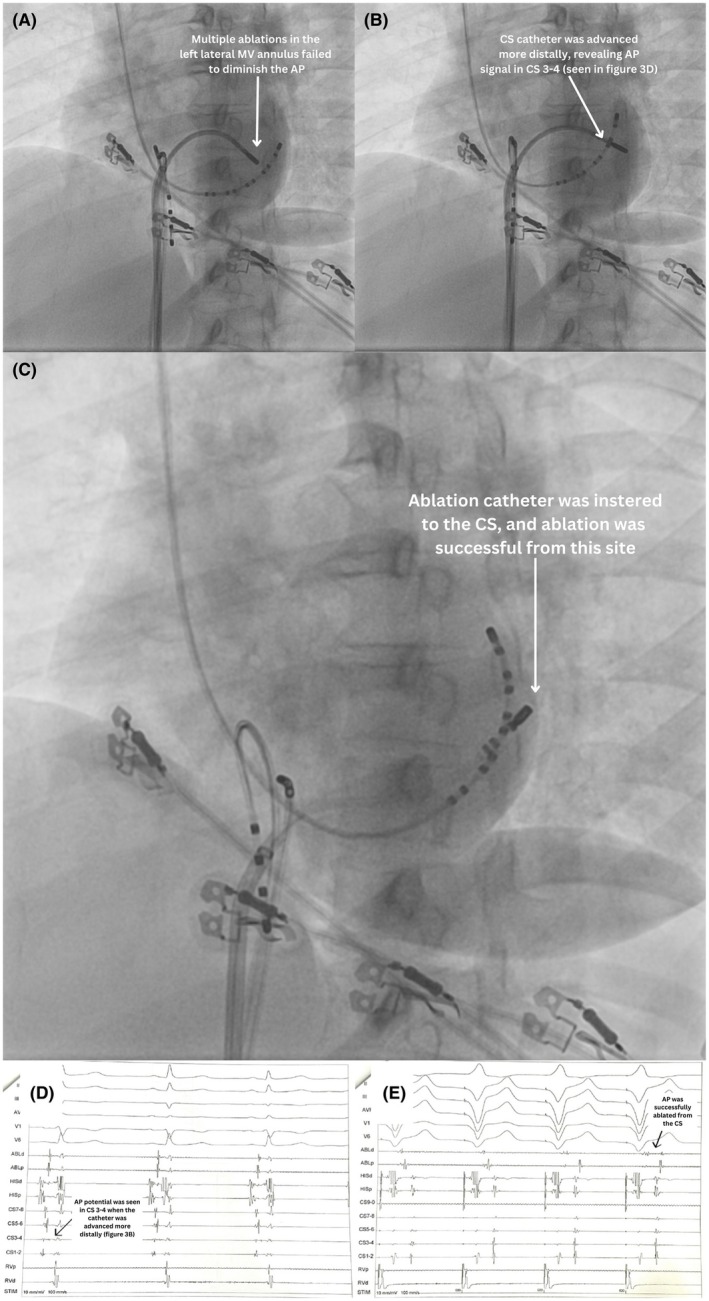
First case: coronary sinus (CS) approach. (A) The ablation catheter was positioned at the left lateral mitral valve (MV) annulus via transeptal puncture; however, multiple ablations at this site failed to eliminate the accessory pathway (AP). (B) The CS catheter was advanced further distally, revealing an AP potential on CS 3–4. (C) The ablation catheter was inserted into the CS, and ablation at this site successfully eliminated the AP. (D) Electrogram (EGM) recorded when the CS catheter was advanced more distally, showing a spike in CS 3–4, likely representing an AP potential. (E) EGM recorded with the ablation catheter positioned in the CS, showing successful elimination of the AP.

A 39‐year‐old female with a history of orthodromic atrioventricular reciprocating tachycardia (AVRT) due to a concealed accessory pathway (AP) in the left lateral region that was confirmed by electrophysiology study was planned for ablation. A transeptal approach was used to access the mitral valve (MV) annulus. Following transeptal puncture, an irrigated ablation catheter was positioned at the left lateral MV annulus (Figure 1A), while diagnostic catheters were placed at the His region and right ventricular apex (RVA). RVA pacing resulted in VA fusion, with the earliest atrial activation recorded distally in the coronary sinus (CS), while high right atrium (HRA) pacing showed antegrade conduction through the AV node. Despite multiple ablations along the left lateral MV annulus, the AP persisted. Further advancement of the CS catheter revealed a sharp potential in CS 3–4, suggesting an AP potential (Figure 1B,D). The observed AP potential conducted antegradely, suggesting that the AP might have had bidirectional conduction properties, given that the patient previously exhibited retrograde conduction during orthodromic AVRT. Radiofrequency (RF) ablation from the CS during RVA pacing using energy 15 W, 45°C, 160–170 ohms directly separated VA in 3 s (Figure 1E). No AP reconnection was observed during PES, burst pacing, or after 30 min of follow‐up. Coronary angiography was not performed prior to ablation within the CS. However, the patient remained asymptomatic and hemodynamically stable during and after the procedure. Furthermore, at 1‐year follow‐up, no coronary artery‐related complications or symptoms were observed, suggesting that the procedure was safely performed.

## 3 SECOND CASE: AORTIC APPROACH

**FIGURE 2:**
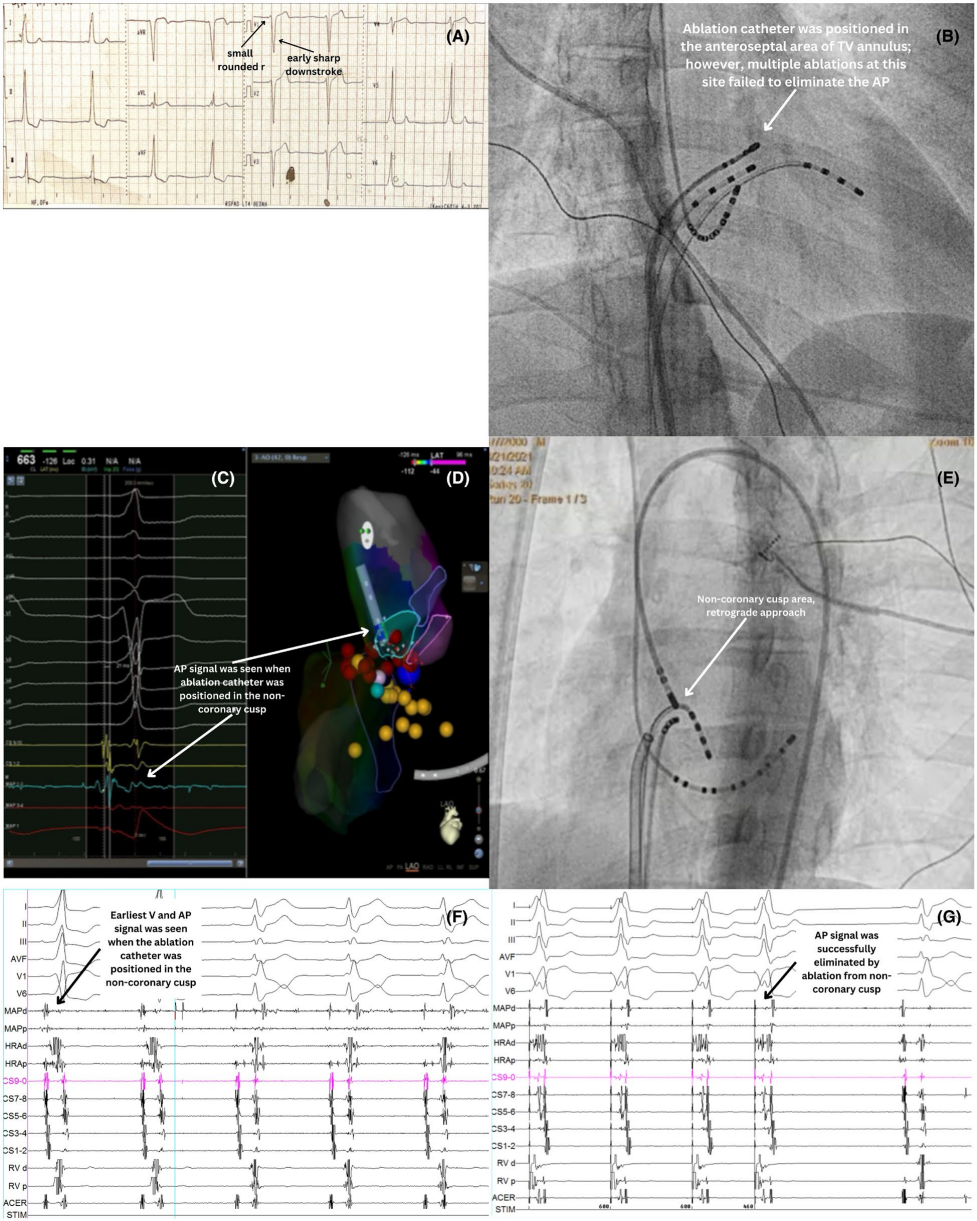
Second case: aortic approach. (A) Initial ECG: The accessory pathway (AP) was localized to the right anteroseptal/nodo‐Hisian area using the EASY‐WPW algorithm (negative QRS in V1, QRS transition ≤V3, and the most positive delta wave in lead II). A small rounded *r* wave and an early sharp downstroke were also observed in the V1 lead, suggesting a paraseptal AP. (B) The ablation catheter was positioned in the anteroseptal area of the tricuspid valve (TV) annulus; however, multiple ablations at this site failed to eliminate the AP. (C) EGM and (D) 3D mapping revealed the AP potential in the noncoronary cusp. (E) Ablation was performed from the noncoronary cusp with retrograde approach. (F) As the ablation catheter was positioned in the noncoronary cusp, the earliest V and AP potential were detected. (G) Ablation was successfully performed in the noncoronary cusp.

A 21‐year‐old male was disqualified from military school due to the incidental discovery of an AP during a medical examination. The AP was localized to the right anteroseptal/nodo‐Hisian area using the EASY‐WPW algorithm (negative QRS in V1, QRS transition < V3, most positive delta wave in lead II) (Figure 2A). Multiple RF titration ablation attempts using a long sheath in this region failed (Figure 2B), and junctional rhythm was repeatedly induced, indicating injury to the His bundle or AV node. The procedure was stopped, but the patient and family insisted on continuing despite the risks. The following day, we proceeded with 3D mapping ablation. Using CARTO 3D mapping, local activation time (LAT) revealed the earliest ventricular activation at the nodo‐Hisian region. However, repeated ablation attempts were unsuccessful, again inducing junctional rhythm, suggesting a possible epicardial component that could not be reached from the endocardium. The retrograde aortic approach was then performed to map the coronary cusps (Figure 2E). A new right bundle branch block (RBBB) was observed during aortic mapping (Figure 2F,G), likely resulting from prior ablation attempts near the His bundle, where repeated energy delivery had induced junctional rhythms. LAT mapping of the right coronary cusp (RCC) and noncoronary cusp (NCC) detected the earliest activation at the NCC (Figure 2C,D,F). Ablation at this site successfully separated AV directly (Figure 2G). No AP reconnection was observed during a 30‐min postablation follow‐up.

## 4 THIRD CASE: RIGHT ATRIAL APPENDAGE APPROACH

**FIGURE 3:**
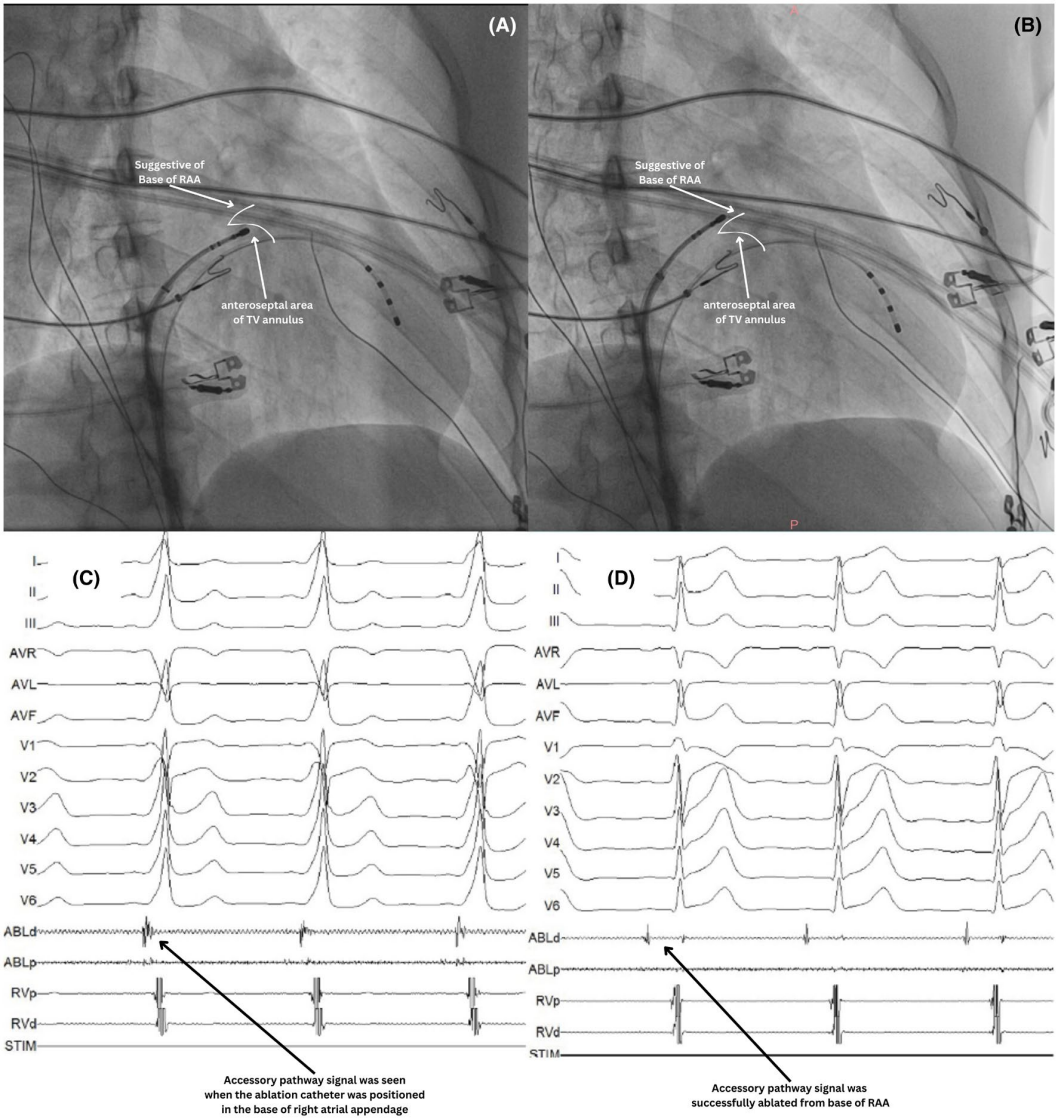
Third case: right atrial appendage (RAA) approach. (A) Multiple ablations from the anteroseptal area of the tricuspid valve annulus failed to eliminate the accessory pathway (AP). (B) Ablation catheter was positioned in the base of the RAA. Ablation from this site was successful. (C) AP potential was seen when the ablation catheter was placed in the base of the RAA. (D) AP was successfully eliminated from the base of the RAA.

A 19‐year‐old male presented with a history of AVRT. The AP was localized to the right anteroseptal/nodo‐Hisian region using the EASY‐WPW algorithm (negative QRS in V1, QRS transition ≤ V3, most positive delta wave in lead II or III) (Figure 3C). Ablation was planned at the right anteroseptal tricuspid valve (TV) annulus (Figure 3A). The HALO catheter recorded a sharp potential suggesting an AP potential at the 11–12 o'clock position in the left anterior oblique 45° view. Pacing from RVA and HRA showed the earliest activation in this region, hence ablation was performed at this site using long sheath and RF titration. However, despite RF ablation, the AP persisted, suggesting an epicardial component unreachable from the endocardium. We notice that AV fusion was shown when the ablation catheter was positioned in the base of right atrial appendage (RAA) using fluoroscopy guidance (Figure 3B,C) with the A potential higher than V. RF ablation from this site (15–20 W for 10–20 s) successfully eliminated the AP in two beats (Figure 3D). Although the ablation was successful, it was unfortunate that we could not perform 3D mapping nor an RAA‐graphy to confirm RAA anatomy. However, the location of the base of RAA was further suggested by the operator's tactile feedback, a key, yet often underappreciated, aspect of electrophysiological guidance. Nonetheless, no AP reconnection was detected during pacing or after 30 min of observation.

## 5 DISCUSSION

Epicardial APs pose unique challenges due to their difficult‐to‐reach locations. One of the most common approaches for epicardial AP ablation, the subxiphoid approach, is relatively challenging compared to endocardial ablation and is also not applicable to all epicardial AP sites such as NCC. This case series demonstrates that some APs with epicardial components can be ablated through a nonsubxiphoid approach, with the approach adjusted based on the pathway's location.

The CS approach is typically effective for ablation of epicardial pathways in the left lateral regions. Haissaguerre et al.^2^ reported a safe and effective RF ablation for left lateral AP using the CS approach when the endocardial approach was unsuccessful, which we adapted in the first case. This is due to the CS myocardial coat connecting both atria and extending to the epicardium of the left ventricle.

An RAA epicardial AP is rare, occurring in less than 0.5% of all cases.^3^ Soejima et al. (1998),^4^ Goya et al. (1999),^5^ and Lam et al. (2000)^3^ all reported successful ablation of right‐sided epicardial pathways originating from the RAA after multiple failed endocardial attempts, which we adopted in the third case. Regarding NCC ablation, this approach is considered effective and safer for para‐Hisian pathways when the endocardial approach fails or risks injuring the His bundle or AV node, as occurred in the second case. Xu et al. (2020)^6^ reported a 91.6% success rate of para‐Hisian AP ablation via the NCC, with zero complications during a 2‐year follow‐up.^6^ The iatrogenic RBBB in our aortic‐approach case (Figure 2F,G) emphasizes the importance of considering alternative ablation strategies for persistent APs with a suspected epicardial component, in order to avoid repeated ablation that may cause injury to the conduction system.

The risk of complications from ablation at these sites remains, including atrial and CS perforation and coronary artery damage. Precise mapping and stepwise ablation approach while keeping the patient alert is crucial for minimizing these risks and identifying any symptoms indicative of these complications. Fortunately, in these three cases, we successfully ablated the AP directly, minimizing the associated risks.

## 6 CONCLUSION

Uncommon epicardial APs can be safely ablated from the CS, aortic cusps, and atrial appendage. Precise mapping of cardiac anatomy and a stepwise ablation approach are critical to minimizing complications. Maintaining patient alertness plays a key role in the early detection of potential complications.

## FUNDING INFORMATION

None declared.

## CONFLICT OF INTEREST STATEMENT

Authors declare no conflict of interests for this article.

## PATIENT CONSENT STATEMENT

This case report is submitted within the knowledge of the patient and his approval. An informed consent letter was provided and signed by the patient. The consent referred to the Committee on Publication Ethics (COPE) guidelines.
